# The Effects of Androgens on T Cells: Clues to Female Predominance in Autoimmune Liver Diseases?

**DOI:** 10.3389/fimmu.2020.01567

**Published:** 2020-07-29

**Authors:** Lara Henze, Dorothee Schwinge, Christoph Schramm

**Affiliations:** ^1^I. Department of Medicine, University Medical Center Hamburg-Eppendorf, Hamburg, Germany; ^2^Martin Zeitz Centre for Rare Diseases, University Medical Center Hamburg-Eppendorf, Hamburg, Germany

**Keywords:** testosterone, androgen, immunity, autoimmunity, androgen receptor, sex-bias, T cell, sex hormones

## Abstract

The immune system responds differently in women and in men. Generally speaking, adult females show stronger innate and adaptive immune responses than males. This results in lower risk of developing most of the infectious diseases and a better ability to clear viral infection in women (1–5). On the other hand, women are at increased risk of developing autoimmune diseases (AID) such as rheumatoid arthritis, multiple sclerosis (MS), systemic lupus erythematosus (SLE), Sjögren's syndrome, and the autoimmune liver diseases autoimmune hepatitis (AIH) and primary biliary cholangitis (PBC) (6). Factors contributing to the female sex bias in autoimmune diseases include environmental exposure, e.g., microbiome, behavior, and genetics including X chromosomal inactivation of genes. Several lines of evidence and clinical observations clearly indicate that sex hormones contribute significantly to disease pathogenesis, and the role of estrogen in autoimmune diseases has been extensively studied. In many of these diseases, including the autoimmune liver diseases, T cells are thought to play an important pathogenetic role. We will use this mini-review to focus on the effects of androgens on T cells and how the two major androgens, testosterone and dihydrotestosterone, potentially contribute to the pathogenesis of autoimmune liver diseases (AILD).

## Androgens in Steady State

The androgenic steroid hormones, testosterone, dihydrotestosterone (DHT), androstenedione, and dehydroepiandrostenone (DHEA) are generated from cholesterol (7). In men, the majority of testosterone precursors (>95%) are produced by Leydig cells in the testes and, to a lesser degree, by the adrenal glands. In women, testosterone precursors are produced by the adrenal glands, the thecal cells of the ovaries, and, during pregnancy, by the placenta (7–10). Metabolism of androgens is complex with testosterone generated from androstenedione in peripheral tissues and the conversion of testosterone into estrogen mediated by the enzyme aromatase in a context and tissue specific manner. Conversion of testosterone into DHT mainly occurs in the liver by the action of 5α-reductase, and DHT cannot be further metabolized to estrogen (11). Sixty-five to 70% of testosterone in blood is bound to sex hormone-binding globulin (SHGB) and 30–35% to albumin, which transport the hormone to target tissues. Only around 0.5–3% of testosterone is found freely in blood (9). Concentrations of bioavailable testosterone can be estimated with total testosterone, SHGB, and albumin serum levels (12).

Interestingly, women show blood androgen levels that are higher than the levels of estrogen. This is due to DHEA produced by the adrenal glands which is subsequently converted to testosterone via androstenedione (8). The levels of total testosterone in women range from 0.35 to 2.94 nmol/l, and there are no significant changes during daytime in testosterone and free testosterone levels (9, 13). In premenopausal women testosterone and free testosterone slightly peak midcycle, but DHT levels do not seem to change during the menstrual cycle (14, 15). With age and after menopause, testosterone levels in women decline, leading to significantly lower levels of testosterone, free testosterone, DHT, and SHGB (13).

In men, testosterone helps to regulate a variety of physiological processes including muscle mass and strength, bone mass, fat distribution, libido, and the production of sperm, red blood cells, and immune cells (11). Due to the complex metabolism of androgens and their tissue and context dependent conversion into estrogen, it is difficult to delineate the action of specific androgens within a given tissue in humans *in vivo*. For example, studies suggest that the effect of testosterone on male bone mass occurs mainly through its conversion to estrogen (16, 17). Serum testosterone levels are significantly higher in men than in women and typically range from 6.2 to 32.1 nmol/l (18). During daytime, a slight decrease in testosterone levels toward the afternoon can be observed (18). Testosterone production in men typically decreases with age to approximately the lower end of the mean levels observed in middle-aged adult men (12, 18–20).

## Androgens Signal Through Cytosolic Androgen Receptor (AR) and Non-Classical Membrane Bound Receptors (mAR)

### Cytosolic Androgen Receptor (AR)

Androgens, including testosterone and DHT, reach their target cells and signal through androgen receptors. In addition to the classical cytoplasmic androgen receptor (AR), androgens can also bind and activate membrane androgen receptors (mAR) (21). DHT binds the AR with a higher affinity and lower dissociation rate than testosterone, while testosterone probably has a higher affinity to the mAR (11). The expression of androgen receptors has been reported in many different tissues, in epithelial and endothelial cells, and in a variety of innate and adaptive immune cells, including human and mouse T cells (22–24).

The classical cytoplasmic AR is a member of the nuclear receptor superfamily and can act as a ligand-dependent transcription factor (25, 26). The human AR gene consists of 8 exons and is located on the X chromosome (27). It has a ligand-binding domain (LBD), a DNA-binding domain (DBD), and an N-terminal domain (NTD) (27). In an unbound state, the AR is residing in the cytoplasm in a complex with chaperons, heat-shock proteins, and cytoskeletal proteins (27, 28). The binding of ligands leads to a conformational change, receptor dimerization, and translocation to the nucleus (29). The NTD affects the transcriptional activity and the DBD permits the binding and recognition of androgen response elements (ARE) on target genes (27). The complex finally disassociates and returns to the cytoplasm (27). AR can also be post-translationally modified through phosphorylation, methylation, or ubiquitination, allowing for ligand-independent modulation of signaling (27, 29, 30).

Next to the regulation of gene transcription, AR interacts with PI3K (phosphoinositide-3-kinase), Src family kinase, and RAS GTPase (27). This interaction affects MAPK/ERK signaling and ERK translocates into the nucleus to affect transcriptional factors leading to adjustment of gene expression involved, e.g., in cell proliferation and survival (27, 28). In a complex but not yet fully elucidated process, mTOR, FOXO1, FOXO3a, HDAC3, STAT3, EGFR, and AKT were shown to be involved in non-genomic AR signaling (27, 28, 31–35).

### Membrane Bound Androgen Receptors (mAR)

The zinc transporter ZIP9 (SLC39A9) has been identified as a membrane bound androgen receptor (mAR), interacting with several kinase pathways such as ERK1/2 and others (36–39). In human prostate cancer cells with overexpressed ZIP9 (PC-3-ZIP9) and breast cancer cells (MDA-MB-468), stimulation with testosterone leads to G proteins being activated, second messenger pathways, and elevation of intracellular free zinc, resulting in initiation of apoptosis and upregulation of pro-apoptotic genes such as BAX, p53, and Caspase-3 (36, 40). In the spermatogenic cell line GC-2, testosterone was shown to induce activation of ERK1/2 and the transcription factors ATF-1 and CREB through Zip9, which interacted with G-protein Gnα11 (38, 39).

The G-protein coupled receptor GPRC6A was suggested as another mAR, which has not yet been reported in a broad range of tissues. *In vitro*, GPRC6A phosphorylates ERK after testosterone stimulation in prostate cancer and bone marrow stromal cells (11, 41). One group showed the involvement of GPRC6A in testosterone production in Leydig cells (42). To our knowledge, however, the expression of GPRC6A in T cells is unknown, reflecting the general lack of knowledge on the role of membrane bound androgen receptors in the immune system.

### Androgen Independent Receptor Signaling

AR signaling can also be induced independently from androgen binding. In prostate cancer cells, IL-6 dependent interplay with AR interferes with the PKA/PKC/MAPK pathway and IL-8 has been shown to promote their AR dependent growth and activation independent of androgens (11, 28, 42–44). Furthermore, IGF-1 stimulated AR phosphorylation, translocation to the nucleus, and upregulation of AR gene expression in myoblast C2C12 cell line (45, 46). These data suggest that inflammation associated changes in the cytokine milieu in an organ affected by autoimmune injury may significantly alter AR signaling. The liver is the central organ of androgen conversion, but so far, the effects of liver inflammation on testosterone metabolism and AR signaling have not yet been explored.

Moreover, the length of the CAG repeat region in exon 1 of the AR gene influences its signaling activity (47–49). Studies in men and women with systemic lupus erythematosus (SLE) and rheumatoid arthritis (RA) demonstrated variable and sex dependent effects of this heritable trait on disease severity and phenotype (50–53).

Overall, the activation and signaling of AR and mAR is complex, and crosstalk between AR transcriptional activity and non-genomic modification of AR- or mAR induced signaling cascades can lead to highly context dependent modification of androgen responses (27).

## Androgens and Human T Cells

AR expression was identified in the majority of innate and adaptive immune cells suggesting that androgens directly modulate the function and development of immune cells. Already in the 1980s, AR expression was reported for human thymocytes (54). Thereafter, AR was found to be expressed on various human and mouse cells of the innate immune system, such as monocytes and macrophages from different tissues, ILC2 progenitors, neutrophils, and mast cells (55–61). In adaptive immunity, AR-expression was shown in human T cells, including CD8+ T cells and CD4+ and splenic CD4+ CD25+ T cells (55, 56, 62–64). In addition to AR, CD4+ and CD8+ T cells were shown to express mAR (65).

The effects of androgens on T cells were studied *in vitro* and by comparing male and female T cells *ex vivo*. It was found that Foxp3 expression, the Treg master transcription factor, was increased in human T cells after DHT treatment *in vitro*, and increased Treg frequencies were reported in men compared to women, and in boys already at the age of eight (66–68). Therefore, androgens may already influence the frequencies of T cells *in vivo* early in life. In adult men, there is a recent report of a negative correlation between CD3+, CD8+, and CD4+ T cells residing in adipose tissue and serum testosterone levels (69). Moreover, upon stimulation of healthy human PBMC with TLR8/9 ligands, secretion of IL-10 in male PBMC was higher than in female PBMC. Upon TLR7 stimulation, IFNα was lower in male PBMC. The amount of IL-10 upon TLR9 stimulation correlated to dehydroepiandrosterone sulfate levels in males, but this study cannot conclude whether these are direct or indirect effects on T cells via dendritic cells (70). Microarray analysis of restimulated T cells showed a higher expression of “pro-inflammatory” genes, such as IFNγ, IL12Rß2, LTß, GNLY, and GZMA in female T cells, while male T cells had a higher expression of IL10, IL5, and IL17A (71). Moreover, healthy male human naïve CD4 cells produced lower levels of IFNγ and had a trend of higher levels of IL-17A upon CD3/CD28 stimulation, possibly through upregulated PPARα and downregulated PPARγ1, and similar results were observed in mice (72–74).

Analysis of men under hormone replacement therapy could give new insight into the effects of androgens *in vivo*, although it is impossible to delineate these *in vivo* effects to single immune cell types such as T cells. Thus, in hypogonadal men a reduction in serum IL1ß and TNF, as well as an increase in IL-10, has been described following testosterone replacement treatment. Whether part of these observed differences related to changes in T cell subpopulations remained speculation (75). In a single case study with one hypogonadal man, an increase in naïve CD4+ CD45Ra+ cells could be observed that could be reverted upon androgen treatment (76). In prostate tissue of BPH (benign prostatic hyperplasia) patients undergoing 5α-reductase type II inhibitor treatment with finasteride leading to reduced intraprostatic DHT levels, a stronger infiltration of CD8+ T cells and higher CCL5 expression was observed (77). Moreover, in a follow-up study, the authors showed *in vitro* that in conditions of low androgen concentrations, CD8+ T cells were able to promote prostate epithelial cell proliferation, possibly through the CCL5/JAK-STAT5/CCND1 pathway (78). After androgen deprivation therapy (ADT) of prostate cancer patients, Wang et al. found enrichment of CD4low HLA-G+ T cells in peripheral blood, besides generally increased CD4+ T cell frequencies (79). In detail, these CD4low HLA-G+ T cells expressed IL-4, IL-17A, and RORγt, indicating an enrichment of IL-4 producing TH17 cells after ADT (79).

Testosterone therapy in transgender individuals offers further possibilities to study the effects of androgens on immune cells *in vivo*. Giltay et al. reported an increase in the IFNγ/IL-4 ratio and TNF production of PBMCs isolated from women undergoing hormone replacement therapy with testosterone. Cells were stimulated with PHA for 36 h and the results indicated increased TH1 differentiation (80). However, as these results contrast some of the above-mentioned studies, they should be further validated and it should be investigated in detail which cell type produced these cytokines.

Taken together, these results provide evidence that androgens influence T cell function and phenotype either directly or indirectly. However, in-depth and comprehensive analyses of direct and context dependent androgen effects on human T cells are lacking.

## Effects of Testosterone on T Cells in Animal Models

Animal models have added to the knowledge on the effects of testosterone on immune cells. Olsen et al. observed a reduced thymus size within 2–4 h after testosterone injection of castrated male mice already in 1998. Mechanistically, increased apoptosis was induced in *in vitro* thymus tissue culture through the AR and reduced percentages of CD4+ CD8+ double positive thymocyte were detected in testosterone treated mice (81, 82). However, several other studies found no direct *in vitro* effect of testosterone on apoptosis of isolated thymocytes (82, 83). A potential explanation for this discrepancy could be that the thymic effects of androgens are mediated by AR expression on thymic epithelial cells (TEC) which are crucial for the negative selection of immature T cells (22, 84, 85). Reduction of androgen levels through castration of mice led to increased numbers of immature triple negative T cells and early T lineage progenitors and a decrease in mature CD4+ and CD8+ single positive cells in the thymus (86). More recently, thymic expression of AIRE (autoimmune regulator) in medullary TECs, which is involved in the thymic selection of T cells by clonal deletion of autoreactive T cells, has been reported to be higher in male human and mouse thymus, possibly induced by the effects of androgens through AR (87). However, ADT by castration of adult male mice did not change TCR diversity but increased the numbers of “naïve” CD44low CD4+ and CD8+ T cells within lymph nodes (88). Additionally, these mice recovered their T and B cells quicker than non-castrated controls after chemotherapy-induced lymphocyte depletion, and these androgen-deprived T cells were more prone to proliferate *in vitro* (88). Taken together, these data suggest that androgens affect T cell maturation and selection within the thymus either directly or indirectly via epithelial cells.

Regarding peripheral mature T cells, it has been shown that DHT treatment in female mice resulted in decreased IL-12 and increased IL-10 production compared to cells from untreated mice following aCD3 stimulation *in vitro*, and this difference was primarily caused by CD4+ T cells (55). Microarray analysis of splenic CD4+ T cells from castrated or control mice showed genes of IFN-signaling and T-helper cell pathways skewed into TH1 differentiation, including upregulation of IFNy, T-bet, and IL-12R (89). Additionally, CXCR3 expression was increased in CD4+ T cells of the castrated group suggesting suppressive effects of androgens on chemokine receptor expression relevant for tissue homing. Along this line, after castration there was an increase in CD3+ cells within lung and prostate tissue. A direct suppressive effect of testosterone on T cells was confirmed by a decrease of IFNy and T-bet expression found in splenic derived CD4+ T cells after treatment with synthetic testosterone *in vitro* (89). Further *in vitro* assays showed a reduction of STAT4 phosphorylation in CD4+ T cells upon androgen and IL-12 stimulation (89).

Confirming direct effects of androgens on mouse T cells, female T cell lines selected in the presence of DHT produced less IFNy and more IL-10 than control cell lines selected without the addition of DHT (63). Splenic derived mouse CD4+ T cells cultured with testosterone-enriched Leydig-conditioned medium showed induction of IL-10 secretion and increased Foxp3 expression, suggesting not only suppression of TH1 cytokines but also an increase in suppressor function of T cells induced by androgens (59, 63). In contrast to some previous reports of a shift toward TH2 cells, Jia et al. found reduced frequencies of TH1 and TH17 cells after *in vitro* DHT and aCD3 stimulation of mouse lymph node cells with no shift toward TH2 cells, possibly through enhanced autophagy in these cells (90). Recently, a reduction of murine *in vitro* TH1 and TH17 differentiation has been demonstrated by aromatase inhibitor treatment in combination with testosterone (91). In addition, visceral adipose tissue (VAT) from male mice showed higher Treg (CD4+ FOXP3+) frequencies then female VAT. The isolated Tregs showed differences regarding phenotype, chromatin accessibility, and transcriptional landscape. In particular, the expression of CCR2 was higher in male VAT Tregs compared to female Tregs. Female mice treated with testosterone showed an increased VAT expression of CCL2, the ligand for CCR2, and IL-6 and IL1ß, which likely stem from innate immune cells (92). These data show that the microenvironment including crosstalk with epithelial and innate immune cells clearly contributes to sex dependent differences observed in T cells.

Taken together, current knowledge suggests that androgens directly or indirectly affect T cell maturation, proliferation, and also their differentiation and cytokine production in mice and adult males. However, little is known on the direct effects of the different androgens on T cells, and specifically on the context dependent cellular and molecular mechanisms involved. Overall, androgens seem to induce a shift from TH1 effector T cells to a more suppressive phenotype. They also seem to enhance regulatory T cells. Clearly, more studies are needed that take into account signaling via classical and non-classical androgen receptors and the context dependent modulation of androgen signaling by an inflammatory microenvironment within tissues.

## Effects of Androgens on T Cells in Autoimmunity

Autoimmune diseases (AIDs) are disorders characterized by an aberrant immune response against self-antigens. There are more than 60 different autoimmune diseases, which pose a major medical and societal challenge. The pathophysiology of most AIDs is complex and includes environmental, genetic, and epigenetic components. Most AIDs present with a strong female predisposition. MS, SLE, and the autoimmune liver disease PBC are among the diseases with the strongest female predominance (Table 1). While many AIDs occur more frequently in women, the course of disease may be more severe in men, exemplified by the worse disease course of male patients with MS or PBC (93, 119). Male PBC patients respond less to treatment with ursodeoxycholic acid and are at increased risk of disease progression and hepatocellular carcinoma development (119, 120). The mechanisms behind these apparent sex differences in disease susceptibility and severity are largely unknown.

**Table 1:** Comparison of autoimmune diseases regarding female to male ratio, knowledge on testosterone serum levels, and therapeutic testosterone application.

**Autoimmune disease**	**F:M ratio**	**Testosterone serum levels**	**Testosterone therapy**	
			**Human**	**Animal model**
Multiple sclerosis (MS)	3:1 (93)	Decreased in male patients (94, 95)	Yes (94, 95)	Yes (96, 97)
Primary biliary cholangitis (PBC)	9:1 (98)	Decreased in female patients in one study (99)	No	Yes (100)
Autoimmune hepatitis (AIH)	3-4:1 (101)	Unknown	No	No (102)
Systemic lupus erythematosus (SLE)	9:1 (103, 104)	Decreased in male and female patients (105)	Yes (106, 107)	Yes (108)
Autoimmune orchitis	Male only	Unknown	No	Yes (109)
Rheumatoid arthritis (RA)	3:1 (110)	Decreased in male and female patients (111, 112)	Yes (113)	Yes (114)
Sjögren's syndrome	14:1 (115)	Decreased in female patients (116)	Yes (116)	Yes (117, 118)

PBC is a rare AID of the liver with a female to male ratio as high as 9:1 and characterized by the presence of anti-mitochondrial antibodies (AMAs), specific antinuclear antibodies (ANAs), and strong HLA associations (98, 121, 122). Immune responses directed against intrahepatic cholangiocytes, leading to the destruction and loss of small bile ducts (ductopenia) and portal inflammation with granuloma formation, are involved in the disease pathogenesis (121–123). In the other classical autoimmune liver disease, AIH, the female to male ratio is 3:1, and patients can present with elevated serum IgG-levels and/or hypergammaglobulinemia, elevated serum transaminase levels, and non-organ specific autoantibodies (101). The target cells of autoimmune attack in AIH are hepatocytes. The human leukocyte antigen alleles (HLA)-DRB1^*^03:01 and HLA-DRB1^*^04:01 are known risk factors for AIH and may also correlate with disease course, but they are not required for AIH development (124). Many lines of evidence support the involvement of CD4+ and CD8+ T cells in both diseases' pathogenesis (122, 125). Studies have investigated the effect of sex hormones on immune cells and how sex chromosomes including X chromosome inactivation affect the sex bias in AIDs (126–130). For example, in PBC an enhanced X monosomy rate within PBMC, possibly T and B cells, compared to healthy women was found, while XCI was random and similar to the controls (131–133). Both PBC and AIH have their age peak of manifestation around menopause, and both show disease modulation by pregnancy with greatly reduced AIH activity during pregnancy and frequent flares after delivery, strongly suggesting the involvement of sex hormones (134–137). Deciphering these mechanisms may lead to novel therapeutic strategies for many of these diseases. We will focus on the studies investigating androgens in the context of autoimmunity and T cells in mouse models and in the human autoimmune liver diseases, AIH and PBC.

There are few mouse models for autoimmune liver inflammation reflecting certain aspects of autoimmune liver diseases and in some of them, a female predominance is observed similar to human disease. In a mouse model of PBC (ARE-Del^−/−^), female mice showed increased serum levels of chemokines, such as MIG and IP-10, as well as increased cytokine levels including TNF, IL-10, and IL-13. They also showed increased expression of interferon Type I and II signaling in the liver compared to the male mice (138, 139). For chronic cholestatic liver inflammation and periductular fibrosis, the Mdr2^−/−^ is a well-established mouse model. Already in 1997 Nieuwerk et al. described a more severe liver pathology in Mdr2^−/−^ female mice compared to male mice which was associated with altered bile salt composition in bile (140). However, the impact of sex hormones on disease development in this model has not yet been investigated. We could recently identify an immunosuppressive effect of testosterone in an antigen dependent and T cell driven mouse model of experimental cholangitis. Cholangitis is induced by the transfer of antigen-specific CD8+ T cells (OT-1) which recognize their ovalbumine peptide antigen on cholangiocytes of recipient mice (100). This model shows a high female predominance. Furthermore, testosterone treatment completely suppressed liver inflammation in female mice and lack of testosterone rendered male mice susceptible to cholangitis development. Mechanistically, we could demonstrate that testosterone suppressed the expression of IL-17A by liver infiltrating lymphocytes and the hepatic expression of the lymphotropic chemokines CXCL-9 and CXCL-10 (100). Similar protective effects of testosterone were also shown in mouse models of MS and murine lupus (96, 97, 141–145). In these models, an influence of sex and androgens on the T cell expression of IFNγ and IL-10 was reported (63, 96, 97). The protective effect of testosterone on EAE development depended on androgen receptor expression and also on age, since older mice were not protected (146). In a mouse model of T cell mediated autoimmune diabetes (NOD mice), a higher *in vitro* CD4+ T cell production of IFNy was observed in female mice and of IL-4 in male mice, which was most prominent in young NOD mice (147). In experimental autoimmune orchitis (EAO), a rat model of a male AID called autoimmune orchitis, testosterone supplementation lead to a reduced incidence of EAO (109). Testosterone treatment decreased the frequencies/numbers of CD4+ T cells and macrophages in the testis, whereas frequencies of Treg populations increased. Furthermore, testosterone treatment resulted in reduced testicular expression of TNF, IL-6, and MCP-1 (CCL2) as well as in reduced secretion of IL-2 and IFNγ of *ex vivo* stimulated mononuclear testicular lymph node cells (109).

It has been difficult to establish mouse models for AIH and few truly represent features of human disease. In one model, xenoimmunization with human antigens (Cytochrome P450 2D6 and formiminotransferase-cyclodeaminase, which are type 2 AIH self-antigens) was used, based on the principle of molecular mimicry. This model showed a higher susceptibility in females compared to males (102, 148, 149). Adoptive transfer of *ex vivo* expanded CXCR3+ Tregs recovered peripheral tolerance and ameliorated disease, but neither castration nor estradiol treatment of these mice had any effect (102, 150). To our knowledge, supplementation with testosterone or DHT was not performed to investigate the suppressive effects androgens might exert.

In humans with autoimmune liver disease, increased serum levels of the proinflammatory cytokines, IFNγ and IL-17 in AIH and PBC patients were reported, while IL-10 was lower than in healthy controls (151). TNF was reduced in the sera of these patients compared to healthy controls, but a recent publication showed an enhanced production of TNF by liver and blood derived CD4+ T cells, with a majority of these cells identified as potentially pathogenic IFNγ co-producers (151). Furthermore, CD4+ T cells of PBC patients revealed increased expression and demethylation of CXCR3, which is the receptor for lymphotropic chemokines produced in inflamed liver (152). Although one older study showed reduced serum levels of testosterone in female PBC patients, it remains unclear whether altered sex hormone levels directly relate to some of the immunological alterations reported above (99).

Data from other AID suggest a role of testosterone in disease pathogenesis. Lower serum levels of testosterone were reported in men with MS compared to age matched healthy men, and testosterone levels seemed to correlate with disease severity (94). Another study suggested lower levels of testosterone in female MS patients compared to female age matched controls (94, 153). Of note, some pilot studies showed disease improvement upon testosterone treatment of male MS patients (94, 95, 154). Also for SLE, lower serum levels of testosterone were reported in affected women compared to age matched healthy women (105, 155, 156). The limitations of these and other studies, summarized in Table 1, are small cohort sizes, and they lack detailed clinical information and the use of now outdated analytical methods. Thus, studies regarding hormone levels in females with AIDs should be interpreted with caution.

Taken together, limited human data and studies using mouse models of autoimmune liver diseases hint to a higher production of proinflammatory cytokines by T cells, but a direct link to sex hormones and, specifically, androgen levels remains unclear. The novel finding of intestinal microbiota associated changes in testosterone serum levels in mice should spark interest in the role of the microbiome for sex differences in autoimmune liver diseases, which are clearly linked to an altered intestinal microbiota (157–159).

## Concluding Remarks

**Figure 1:**
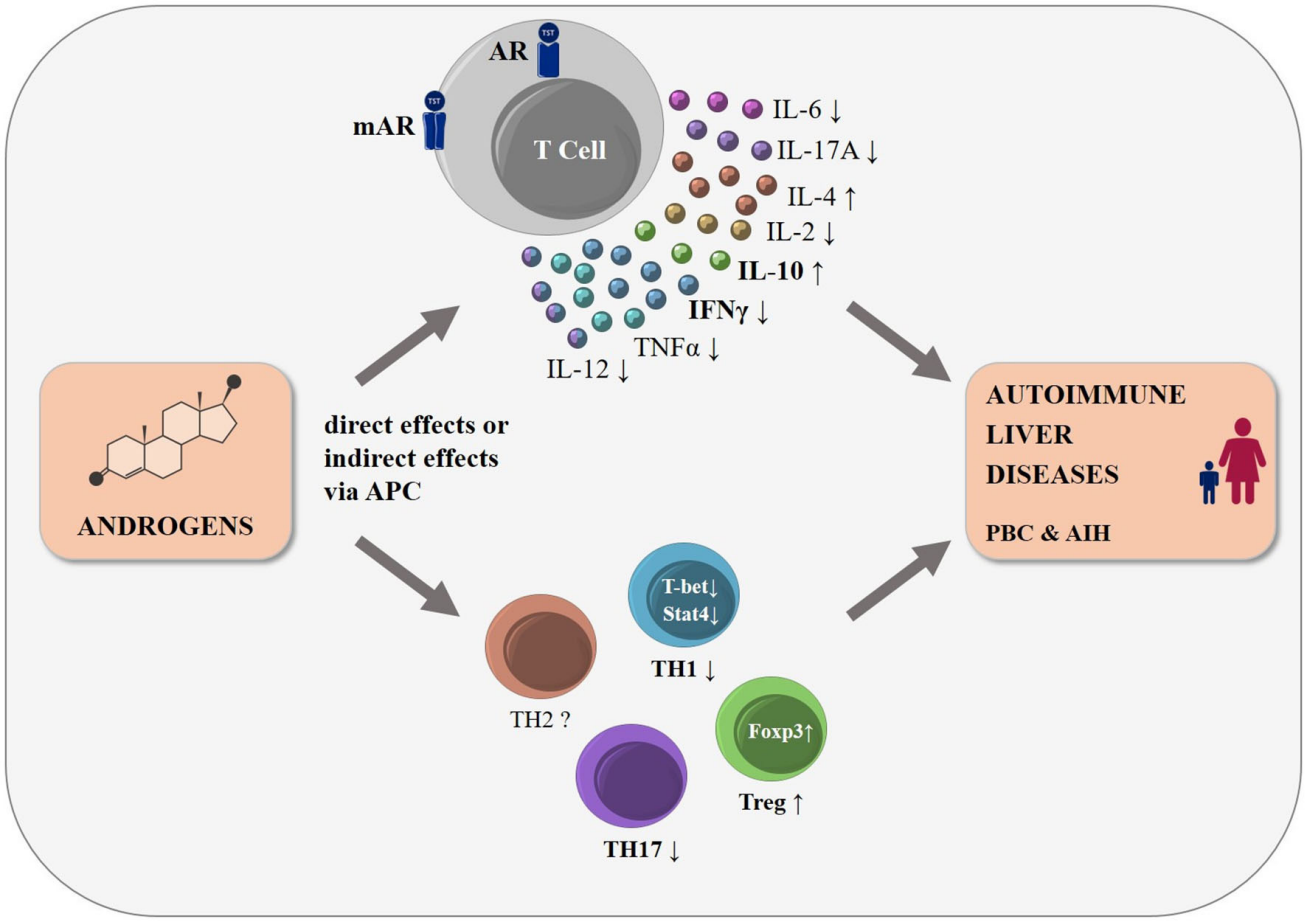
The influence of androgens on T cell function and differentiation: schematic representation. Many autoimmune diseases, including the autoimmune liver diseases PBC and AIH, show a strong female predominance. Androgens modulate T cell development already in the thymus, mainly by altering thymic epithelial cell function (not shown). Human and mouse T cells express cytosolic (AR) and membrane bound androgen receptors (mAR). Androgens lead to changes in cytokine expression in T cells either directly or indirectly via antigen presenting cells, with a shift to a decreased pro-inflammatory cytokine expression, such as IFNγ and TNF, and an increased secretion of anti-inflammatory cytokines such as IL-10 and IL-4. Androgens were reported to reduce TH1-, TH17-, and to increase Treg-differentiation, while changes in other T cell subpopulations (e.g., TH2 cells) remain less clear. We postulate that these androgen-induced effects may influence the incidence and disease course of the T cell driven autoimmune liver diseases, PBC and AIH.

The mechanisms behind the sex differences observed in the autoimmune liver diseases PBC and AIH, specifically the female predominance and worse disease course in male PBC patients, remain largely unknown. Emerging evidence mainly from murine studies suggests immunosuppressive effects of androgens on T cells (Figure 1). More studies are needed to decipher signaling pathways involved in T cells upon androgen stimulation including the classical and non-classical androgen receptors and their modulation by the local microenvironment. Understanding the effects of androgens on immune cells may pave the way for novel treatment strategies for autoimmune liver diseases.

## Author Contributions

LH, DS, and CS designed and wrote the manuscript. All authors contributed to the article and approved the submitted version.

## Conflict of Interest

The authors declare that the research was conducted in the absence of any commercial or financial relationships that could be construed as a potential conflict of interest.

## Abbreviations

AID: autoimmune diseases
SHGB: sex hormone-binding globulin
DHT: dihydrotestosterone
AR: androgen receptor
mAR: membrane bound androgen receptors
LBD: ligand-binding domain
DBD: DNA-binding domain
NTD: N-terminal domain
ARE: androgen response elements
PI3K: phosphoinositide-3-kinase
CREB: cAMP response element-binding protein
Treg: regulatory T cell
BPH: benign prostatic hyperplasia
ADT: androgen deprivation therapy
AIRE: autoimmune regulator
VAT: visceral adipose tissue
MS: Multiple sclerosis
PBC: primary biliary cholangitis
AIH: autoimmune hepatitis
SLE: systemic lupus erythematosus
EAE: autoimmune encephalomyelitis
AMAs: anti-mitochondrial antibodies
ANAs: antinuclear antibodies
EAO: experimental autoimmune orchitis.
